# Altered Features of Vimentin-containing Cells in Cerebrum of Tg(SOD1*G93A)1Gur Mice: A Preliminary Study on Cerebrum Endogenous Neural Precursor Cells in Amyotrophic Lateral Sclerosis

**DOI:** 10.7150/ijbs.33461

**Published:** 2019-11-01

**Authors:** Chunyan Tang, Lei Zhu, Qi Zhou, Menghua Li, Yu Zhu, Zhenzhen Xu, Yi Lu, Renshi Xu

**Affiliations:** 1 Department of Neurology, Jiangxi Provincial People's Hospital, Affiliated People's Hospital of Nanchang University, Nanchang 330006, Jiangxi, china; 2 Department of Neurology, First Affiliated Hospital of Nanchang University, Nanchang 330006, Jiangxi, China

**Keywords:** Amyotrophic lateral sclerosis, Vimentin, Neural precursor cells, Astrocyte, Cerebrum

## Abstract

Vimentin-containing cells (VCCs) are potential neural precursor cells in central nervous systems, Thus, we studied the alteration of VCCs proliferation, differentiation and migration in the cerebrum during different stages of Tg(SOD1*G93A)1Gur mice. It aims to search potential ways regulating the proliferation, differentiation and migration of endogenous VCCs, to enhance their neural repair function and to cure or prevent from the development of ALS. We observed and analyzed the proliferation, differentiation and migration of VCCs in different anatomic regions and cell types of cerebrum at different stages including the pre-onset (60-70 days), onset (90-100 days) and progression (120-130 days) of wild-type (WT) and Tg(SOD1*G93A)1Gur mice using the fluorescent immunohistochemical technology. Results showed that VCCs in the cerebrum were mostly distributed in the ventricular system, periventricular structures, the hippocampus and the cerebral cortex in WT mice. VCCs significantly reduced in the motor cortex and the cingulate cortex in Tg(SOD1*G93A)1Gur mice. All vimentin expressed in the extranuclear and almost all VCCs were astrocytes in WT mice and Tg(SOD1*G93A)1Gur mice. There were no significant difference in the number of Brdu and nestin positive cells in left and right brains of WT mice and Tg(SOD1*G93A)1Gur mice in the period of 60-130 days. Our data suggested that there existed extensively NPCs in the cerebrum of adult mice. In ALS-like Tg(SOD1*G93A)1Gur mice, VCCs in the motor cortex, the olfactory cortex and the cingulate cortex showed that no any proliferation and redistribution in neural cells of VCCs in the cerebrum occurred in all stages of ALS, might migrate to damaged regions.

## Introduction

Amyotrophic lateral sclerosis (ALS) is an irreversible neurodegenerative disorder. Its pathological characterizations are the progressive and selective motor neuron degeneration. Featured pathological alterations mainly consist of the coexistence of upper and lower motor neuron damage, which result in the progressive paralysis of voluntary muscle in upper, lower limbs and/or laryngopharyngeal regions. This disease usually accompanies by pathological signs and/or the tendon reflex hyperfunction. Subsequently, this disease involves in the atrophy of respiratory muscle, ultimately dies from the respiratory failure because of the paralysis of respiratory muscle 1, 2. The typical ALS patient often dies in 3-5 years after the onset of symptoms. Majority of ALS patients are sporadic ALS (sALS). Only approximate 10% of cases are familial ALS 3. The pathogenesis about ALS still isn't completely known now 4. ALS is a disaster disease for middle and senior populations, seriously affects their health and life quality, largely reduces their lifetimes 1-3. To date, no any effective treatment can cure or prevent from ALS 5.

According to present studied reports, the pathogenesis of ALS may be associated with multiple elements, among them, environmental and genetic factors are considered as be two main elements 6, 7. Environmental factors consist of endogenous and exogenous environmental conditions. Current known exogenous environmental factors are to point environmental pollutants, such as chemical pollutants (Pesticide, chemical fertilizer, herbicide) 8, factory hazardous air pollutants 9, the electrical trauma 10, the continuous high levels exposure of some heavy metals 11-14 and the chronic virus and bacterial infection 15. The endogenous environmental factors are to point that the unbalance of cellular environments. Potential factors reported by researchers in the past time majorly involve in the excessive production and/or the eliminate reduction of oxidative free radicals 16, 17, the breakout of calcium homeostasis 18-20, the excessive release and/or the reuptake disorder of excitatory amino acid neurotransmitters 21-23, the abnormal inflammatory responsible 24, 25, the autophagy and/or apoptotic excess of neural cells 26-29, and the abnormality of autoimmune response 30. Based on the potential pathogenesis of ALS, a series of studies searching cured or preventive ways of ALS were conducted in the past time, however, no any effective methods were found 31.

Apart from above mentioned cured and/or prevented methods targeted to pathogenic factors of ALS, the transplantation of neural stem cells (NSCs) was thought to be a potential and hopeful way in the treatment of ALS 32, 33. Major transplanted stem cells consist of NSCs such as neuron stem cells 34, neural glial stem cells and other derived stem cells like bone marrow mesenchymal stem cells, embryo stem cells and amniotic stem cells 35, 36. Transplanted ways mainly consist of intravenous 37, intraventricular 38, subarachnoid and local stereotactic injection. It is so pity that the exogenous transplantation in the treatment of central nervous system (CNS) diseases including ALS only obtained some insignificant effects after years of painstaking work 32, 33. Major reasons include: One is that transplanted cells used to replace damaged neuron cells can't reconstruct the comprehensive synaptic junction and transmit the physiologic signal, although they can proliferate and differentiate at the short time in transplanted regions. Another is that transplanted cells used to produce neurotrophic and neural growth factors only generate the relatively limited amount in the very short time. Current transplanted technologies of exogenous stem cells in CNS aren't enough to repair the damage of CNS. Furthermore, the exogenous transplantation of stem cells will bring a lot of side effects that it is very difficult to conquer to us, and its fee is very expensive for patients 32, 33. Therefore, the transplantation of exogenous stem cells in the treatment of CNS diseases could not obtain the satisfactory effect because of their serious insufficiencies, which bring the transplantation of exogenous NSCs get into a dilemma.

In recent years, a series studied results that neural precursor cells (NPCs) or NSCs exist in the adult CNS were reported one after another 39-44, and partial results revealed that NPCs or NSCs could proliferate, differentiate and migrate to damage regions in Alzheimer's disease 42 and partially repair the deficit of neural function in some pathological conditions including ALS 40, Parkinson's disease 39 and spinal cord injury 41, but the repairing ability wasn't enough to recover the neural function. In view of the fact, the promotion of endogenous NPCs or NSCs in CNS might be a promising way in the repair of CNS including ALS. For that, the elucidation of mechanism about the proliferation, differentiation and migration of NPCs or NSCs in CNS at the pathological condition of ALS is the important and preliminary study, which searches ways enhancing the repairing ability of endogenous NPCs or NSCs through modulating their proliferation, differentiation and migration function.

Vimentin-containing cells (VCCs) in CNS are the potential NPCs or NSCs 45-48. In this study, we intended to find possible features of their distribution, proliferation, differentiation and migration through observing and analyzing altered features of VCCs in the cerebrum in ALS-like Tg(SOD1*G93A)1Gur mice, provide some evidences for modulating them in the treatment of ALS.

## Animals and Methods

### Animals

The line of Tg(SOD1*G93A)1Gur mice (Jackson laboratory, Bar Harbour, Maine) was maintained by mating transgenic males with C57BL/6 wild-type (WT) females in the neurological lab of First Affiliated Hospital of Nanchang University. Mice were identified whether or not they were positive Tg(SOD1*G93A)1Gur mice using the PCR method of genomic DNA derived from the mice tail. Used primers were: The IL-2 forward primer 5'-CTA GGC CAC AGA ATT GAA AGA TCT-3', the IL-2 reverse primer 5'-GTA GGT GGA AAT TCT AGC ATC ATC C-3', the hmSOD1 G93A forward primer 5'-CAT CAG CCC TAA TCC ATC TGA-3', the hmSOD1 G93A reverse primer 5'-CGC GAC TAA CAA TCA AAG TGA-3'. Amplification conditions were: 94 °C degeneration for 3 seconds, 60 °C annealing for 1 minute and 72 °C extension for 1 minute, total 35 cycles. Experimental mice were euthanized at the time points of pre-onset (60-70 days), onset (90-100 days) and progressive (120-130 days) stage 49-52. At experimental time points of different disease stages, gastrocnemius muscles of abnormal limbs were performed the routine muscle biopsy, observed changes of muscle structures in the light microscope, and further detected the paralysis scale of limb muscle and identified disease stages of pre-onset, onset and progression 49-52. Above described protocols were similar with that of our previous published paper 52. All animal studies and experiments were conducted in accordance with the Guide for the Care and Use of Laboratory Animals of China and were reviewed and approved by the ethics committee for animal care and use of First Affiliated Hospital of Nanchang University, China.

### Fluorescent immunohistochemical stain of cerebrum

WT and Tg(SOD1*G93A)1Gur mice were anesthetized and perfused with 20 ml 0.9% saline and 40 ml 4% 1x PBS (pH 7.5) paraformaldehyde (PFA) at room temperature. The cerebrum was excised and placed in 4% PFA buffer overnight, then incubated in 20% 1x PBS (pH 7.5) sucrose for 1 days and 30% 1x PBS (pH 7.5) sucrose for 3 days, followed by embedding in the optimal cutting temperature compound (OCT). Cerebrum tissues were coronally successively cut 12 μm of sections on a Leica cryostat and collected on Superfrost Plus slides. For the fluorescent immunohistochemical stain of cerebrum sections, all sections were permeabilized by 0.2% Triton X-100, blocked by 10% 1x PBS goat serum after rehydrated in 1x PBS (pH 7.4), then incubated by following antibodies (Vimentin 1:100, Santa cruz biotechnology Inc; Nestin 1:50, Abcam (Hong Kong) Ltd; NeuN, 1:250; GFAP, 1:1000; Oli-2, 1:100 and Ox-42, 1:100, Abcam (Hong Kong) Ltd.) at 4 °C overnight, subsequently washed 6 times using 0.2% 1x PBS Triton X-100 for each 5 minutes, incubated using secondary antibodies (Donkey anti goat, 1:250, donkey anti rabbit, 1:200) conjugated to the green fluorescence or/and the red fluorescence (Rhodamine) for 2 hours at room temperature. For the 4',6-diamidino-2-phenylindole (DAPI) stain (Blue fluorescence), extensively washed 5 times for 5 minutes each times, mounted using antifade medium, then observed and took pictures under a Nikon E800 fluorescent microscope with a spot digital camera (Diagnostic Instruments, Sterling Heights, MI, USA) and Photoshop software (Adobe Systems, San Jose, CA, USA). The multiple labeled fluorescent histochemistry conjugated to anti vimentin, NeuN, GFAP, Oli-2 or Ox-42 antibodies and DAPI was used to investigate the proliferation and the differentiated cell types of VCCs.

### Brdu administration and staining of cerebrum

To test neurogenesis in our experiments, we injected Tg(SOD1*G93A)1Gur mice and littermate controls with Brdu (50 mg/kg, intraperitoneally) twice on the same day (At 12 hours intervals). The Brdu concentration was 10 mg/ml, total injections were 2 times. After the Brdu injection, mice were continued to feed for 7 days, then euthanized them, collected the sample of cerebrum, fixed, embeded and sliced under the no light condition. Other protocols were the same with the above described fluorescent immunohistochemical staining.

### Analysis of positive cells

The analysis of positive cells was performed by counting amount of positive cells in the cerebrum at 200 magnifications in 10 sections and calculating the positive cells sum of all 10 sections, then the sum was divided by the section number, 3 mice per group were used, the averaged amount or percent was used for the quantitative analysis.

### Statistical analyses

All experimental data were expressed as mean ± SD. Specific comparison between the control and individual experiment was analyzed by ANOVA and Student's t-tests, P < 0.05 was considered as the statistically significant.

## Results

### Distributed features of VCCs in the adult cerebrum of WT mice

**Figure 1:**
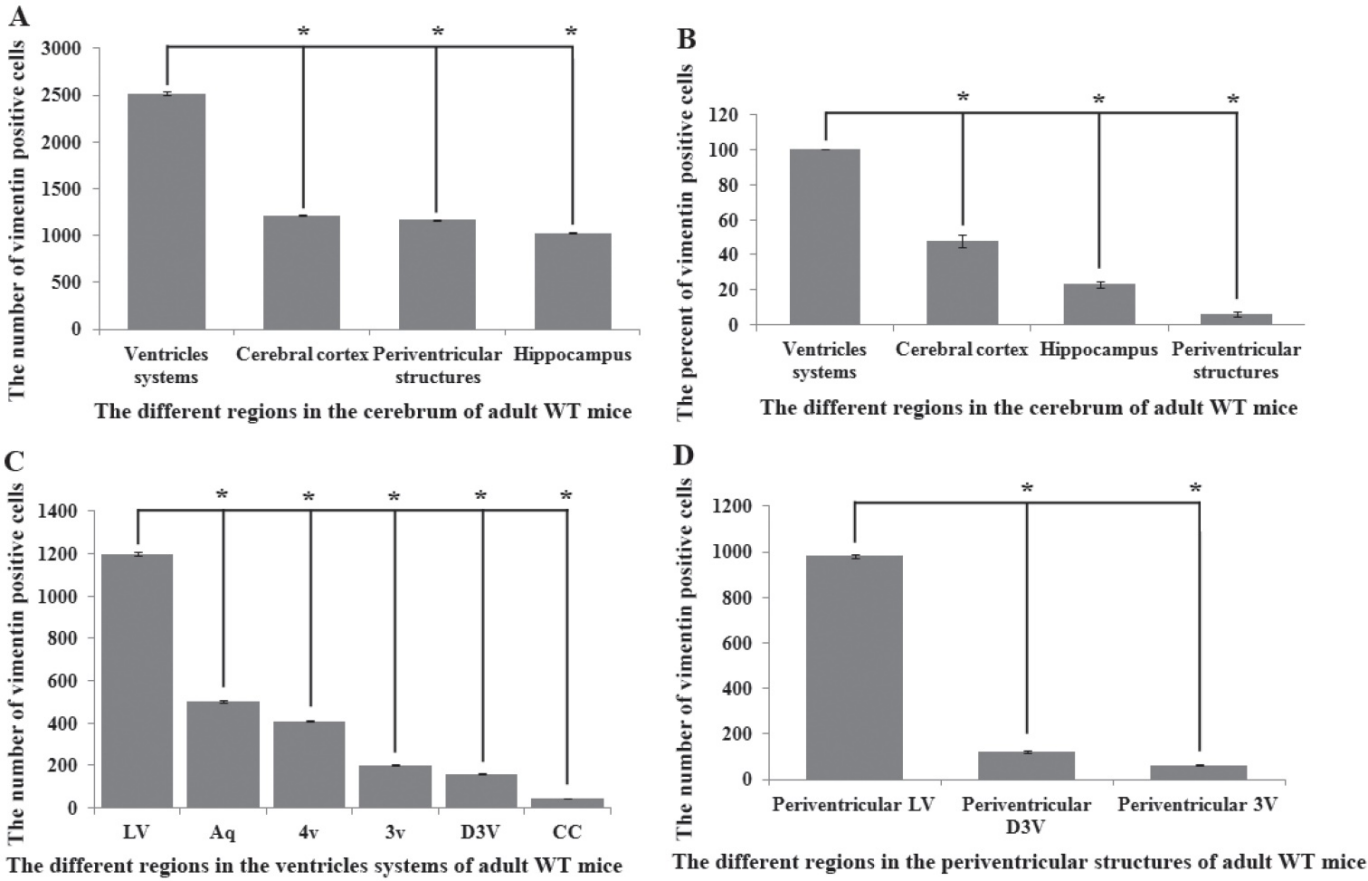
** Distribution features of vimentin positive cells (VPCs) in different regions of cerebrum in adult wild-type (WT) mice. A** The number of VPCs in different regions of cerebrum in adult WT mice. VPCs in the cerebrum mainly distributed in the ventricular system, periventricular structures, the hippocampus and the cerebral cortex, the number of ventricular system > periventricular structures > the hippocampus > the cerebral cortex. **B** The percent (Density) of VPCs in different regions of cerebrum in adult WT mice. The percent of VPCs in the ventricular system > the cerebral cortex > the hippocampus > periventricular structures. **C** The number of VPCs in different regions of ventricular system in adult WT mice. The number of VPCs in LV > Aq > 4V > 3V > D3V > CC. **D** The number of VPCs in different regions of periventricular structures in adult WT mice. The number of VPCs in the periventricular LV > the periventricular D3V > the periventricular 3V.

**Figure 2:**
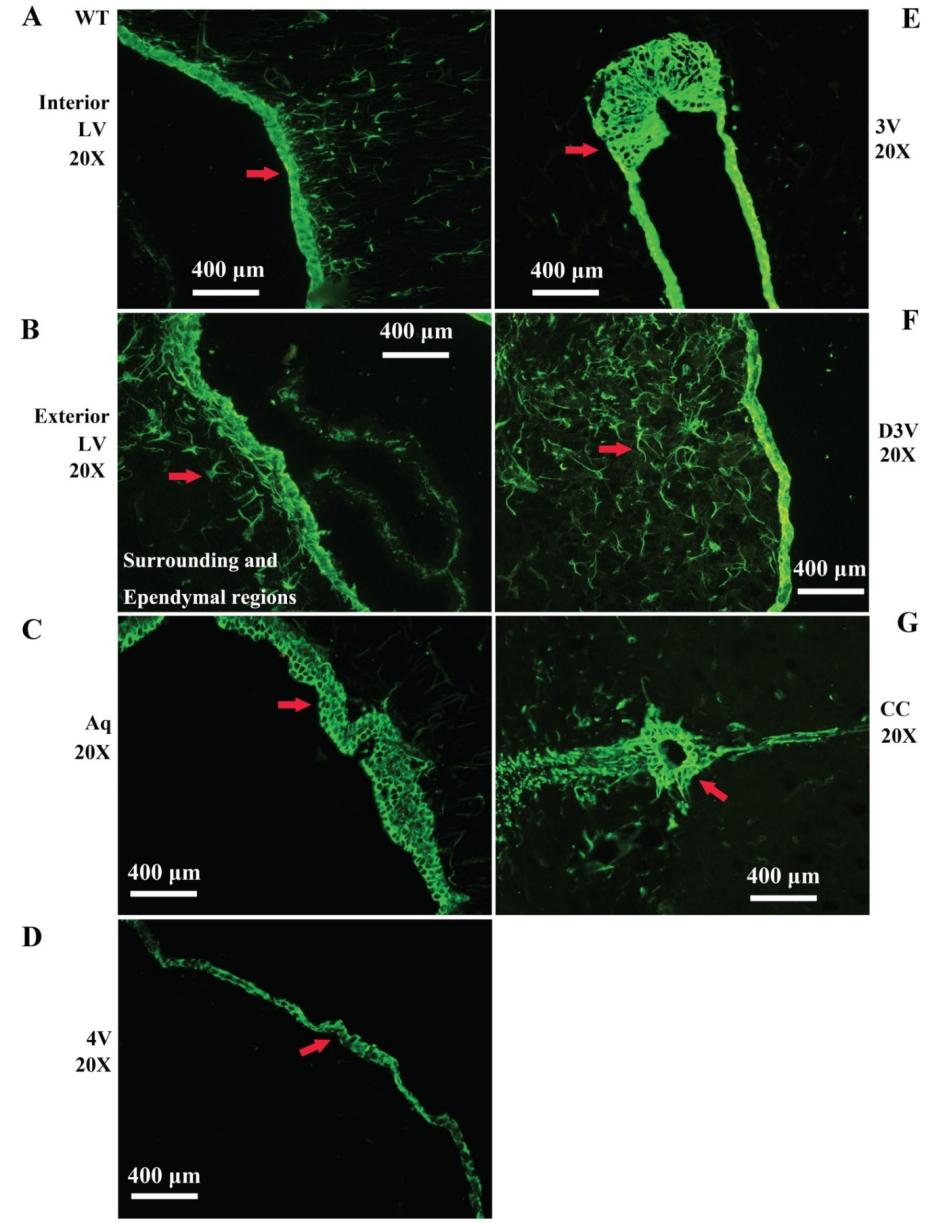
** Representative images of VPCs distribution in different ventricular systems in adult WT mice. A, B** The VPCs distribution in LV. The VPCs in LV mainly distributed in the interior (**A**), the exterior and the surrounding and Ependymal regions (**B**). **C** The VPCs distribution in Aq. VPCs in Aq mainly distributed in the ependymal region. **D** The VPCs distribution in 4V. VPCs in 4V mainly distributed in the ependymal region. **E** The VPCs distribution in 3V. VPCs in 3V mainly distributed in the ependymal region. **F** The VPCs distribution in D3V. VPCs in D3V mainly distributed in the ependymal region. **G** The VPCs distribution in CC. VPCs in CC mainly distributed in the ependymal region. The red arrow marked the positive cell, bar = 400µm.

**Figure 3:**
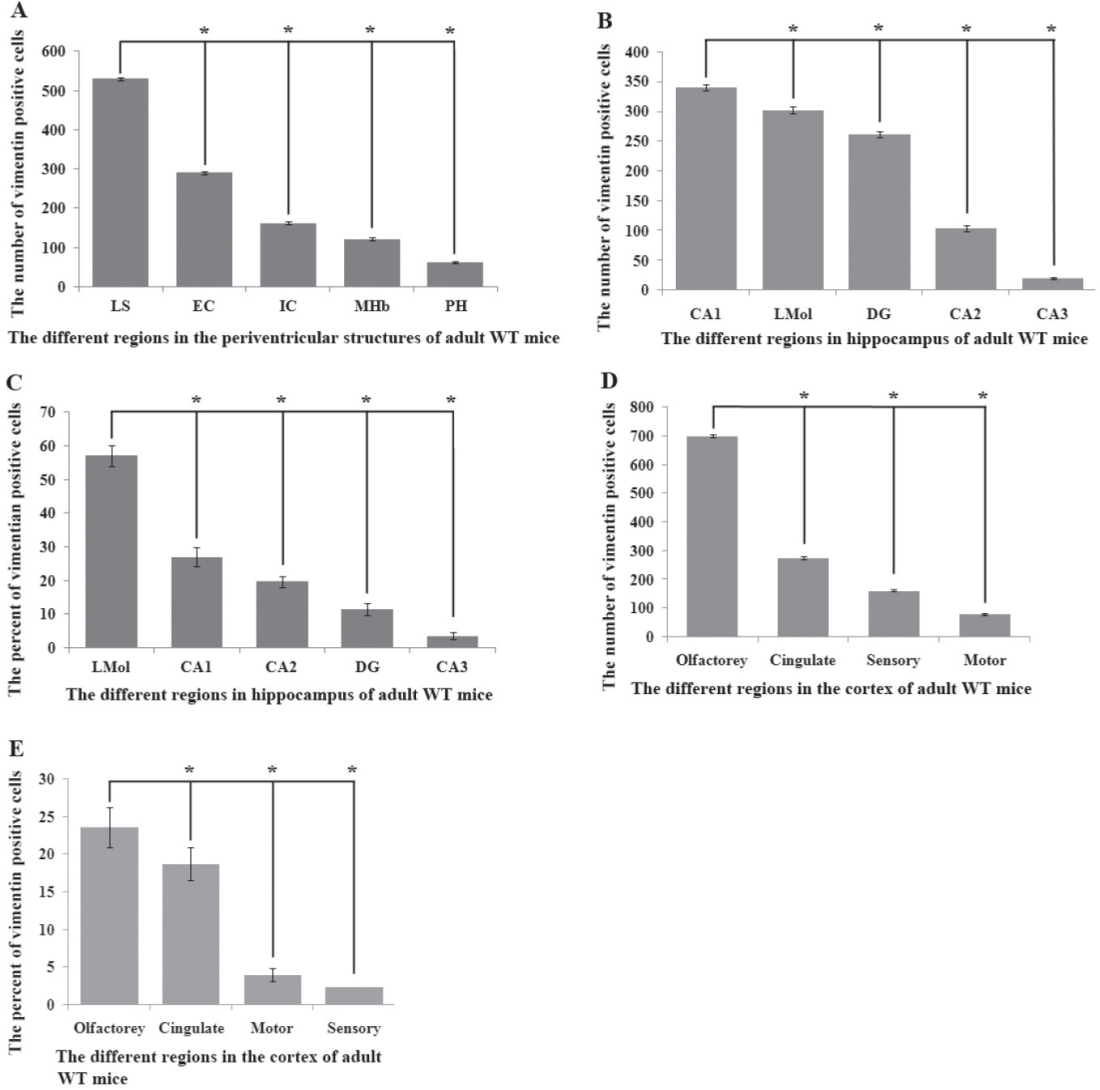
** Distribution features of VPCs in different regions of periventricular structures, the hippocampus and the cerebral cortex in adult WT mice. A** The number of VPCs in different regions of periventricular structures in adult WT mice. The number of VPCs in LS > EC > IC > MHb > PH. **B** The number of VPCs in different regions of hippocampus in adult WT mice. The number of VPCs in CA1 > LMol > DG > CA2 > CA3. **C** The percent of VPCs in different regions of hippocampus in adult WT mice. The percent of VPCs in LMol > CA1 > CA2 > DG > CA3.** D** The number of VPCs in different regions of cerebral cortex in adult WT mice. The number of VPCs in olfactory > cingulate > sensory > motor cortex. **E** The number of VPCs in different regions of cerebral cortex in adult WT mice. The percent of VPCs in olfactory > cingulate > motor > sensory cortex.

**Figure 4:**
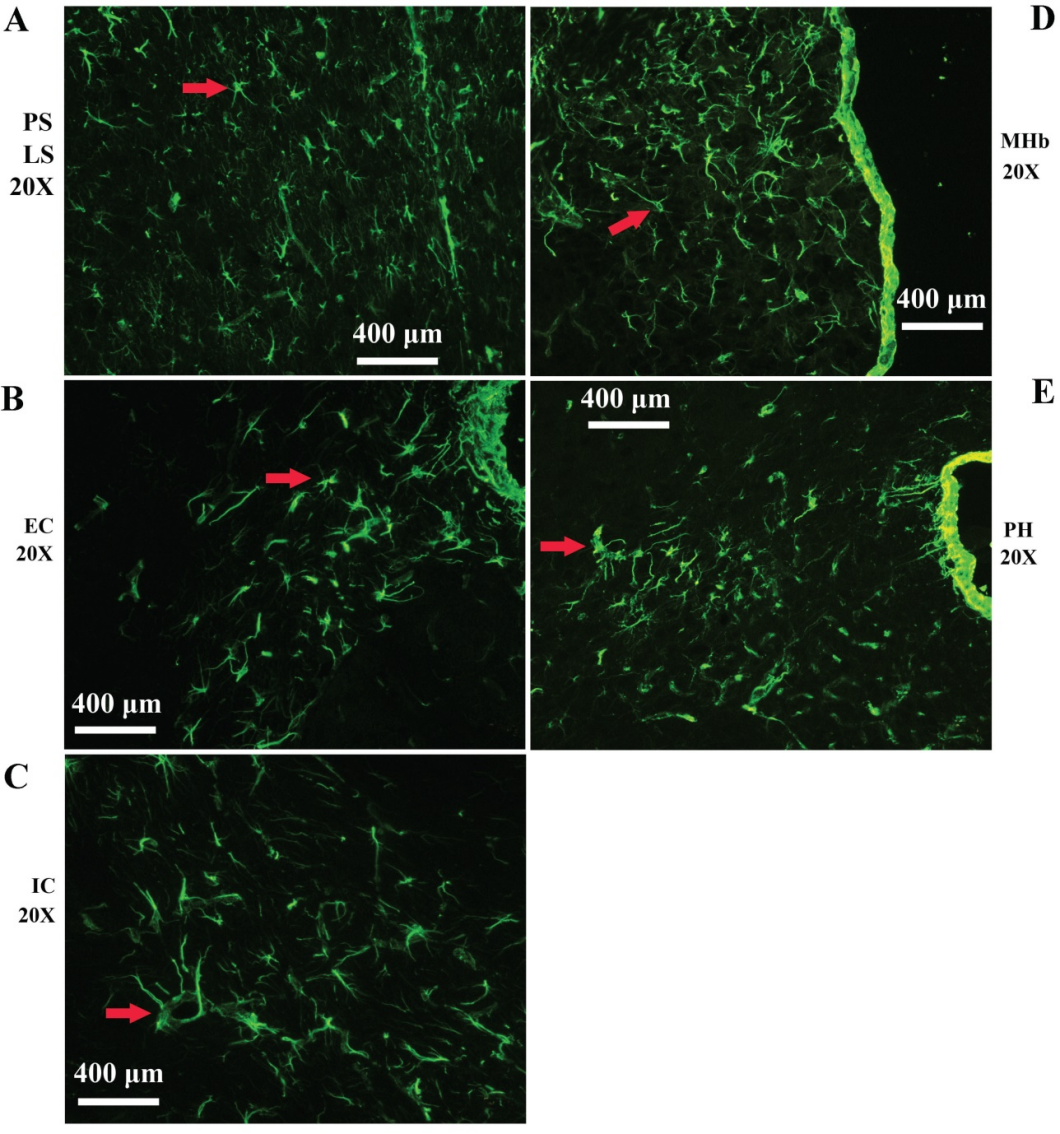
** Representative images of VPCs distribution in different regions of periventricular structures in adult WT mice. A** The VPCs distribution in LV. VPCs in LV mainly distributed in LS. **B** The VPCs distribution in EC. **C** The VPCs distribution in IC. **D** The VPCs distribution in MHb. **E** The VPCs distribution in PH.

**Figure 5:**
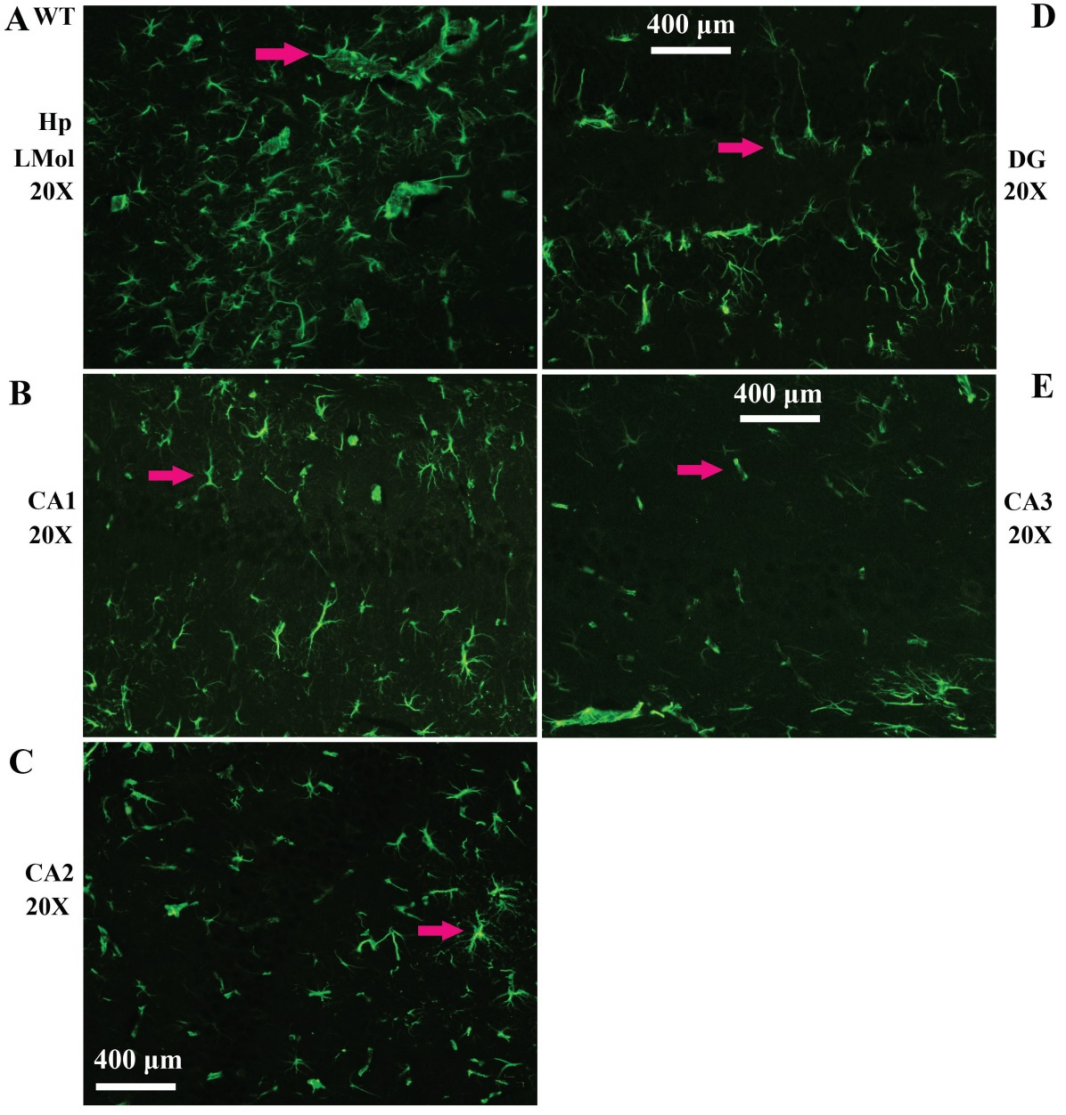
** Representative images of VPCs distribution in different regions of hippocampus in adult WT mice. A** The VPCs distribution in LMol. **B** The VPCs distribution in CA1. **C** The VPCs distribution in CA2. **D** The VPCs distribution in DG. **E** The VPCs distribution in CA3.

**Figure 6:**
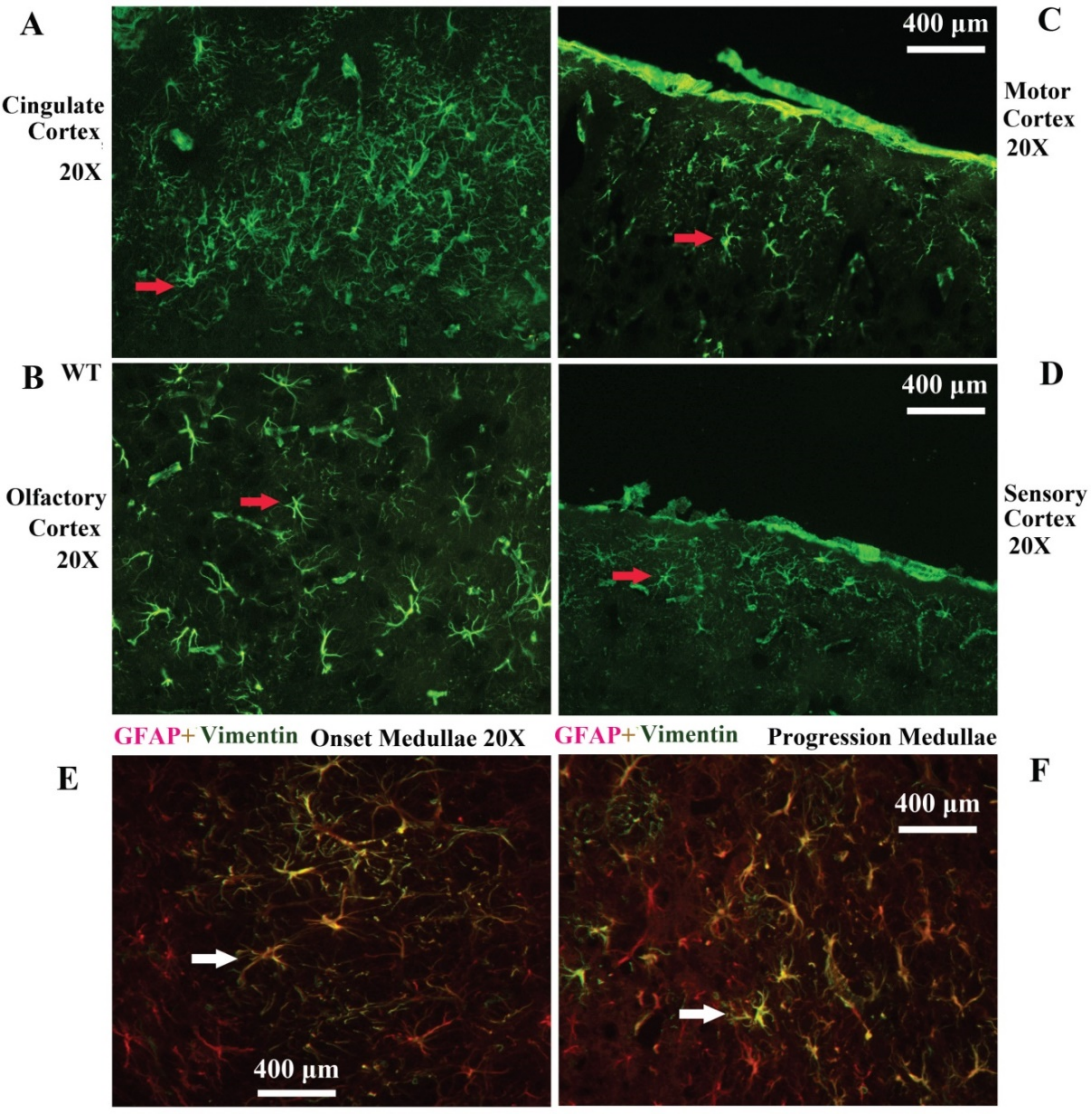
** Representative images of VPCs distribution in different regions of cerebral cortex in adult WT mice. A** The VPCs distribution in the cingulate cortex. **B** The VPCs distribution in the olfactory cortex. **C** The VPCs distribution in the motor cortex. **D** The VPCs distribution in the sensory cortex. Representative Images of vimentin and GFAP double labeled immunofluorescent staining in the cerebrum **(E, F)**. **E** Representative image of vimentin and GFAP double labeled immunofluorescent staining in the medullae at the onset stage of Tg(SOD1*G93A)1Gur mice. **F** Representative image of vimentin and GFAP double labeled immunofluorescent staining in the medullae at the progression stage of Tg(SOD1*G93A)1Gur mice. All vimentin positive cells were labeled by GFAP, which indicated that all vimentin cells were astrocytes.

**Figure 7:**
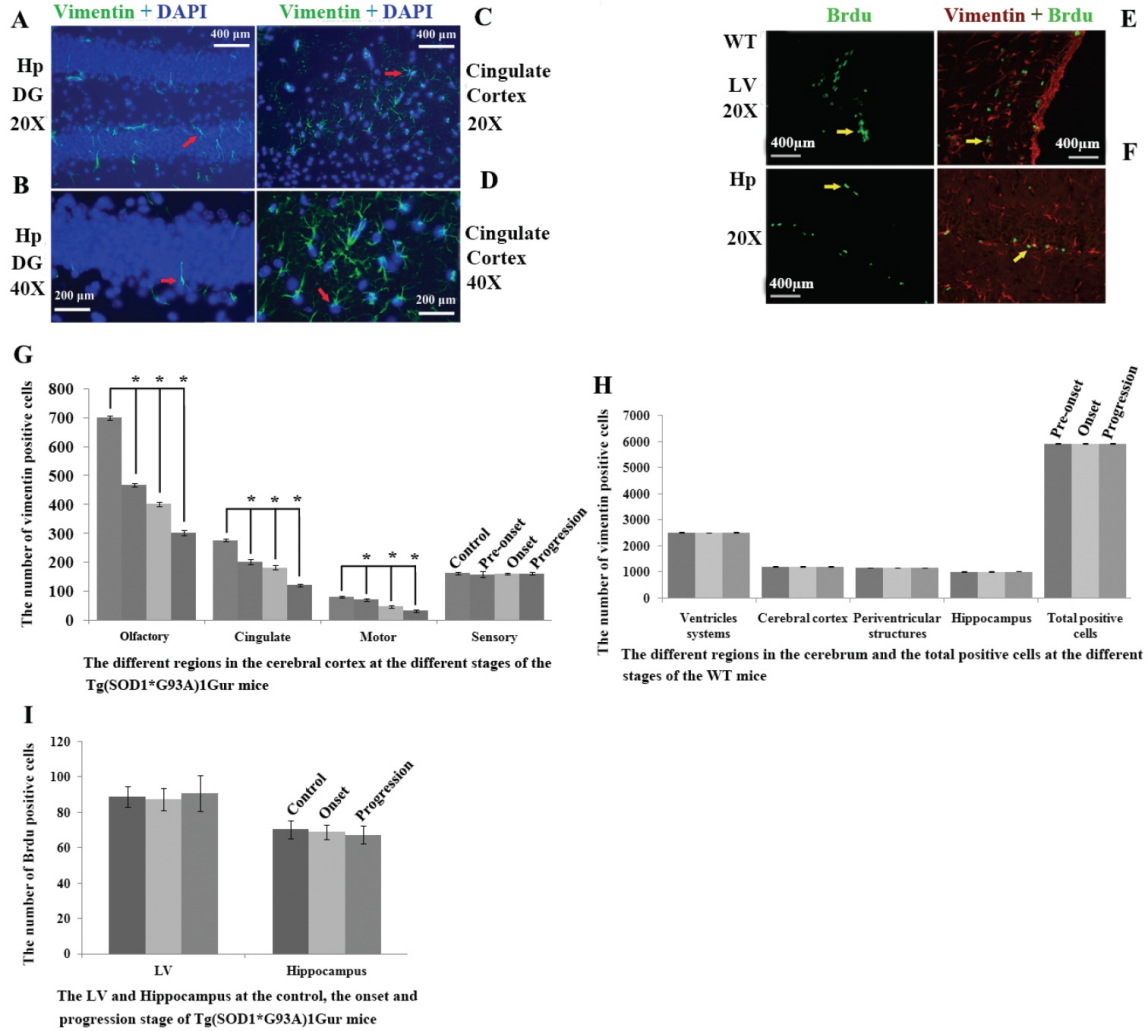
** Representative images of vimentin expression in neural cells of cerebrum. A** Representative images of vimentin expression in neural cells of hippocampus DG at amplifying 200.** B** Representative images of vimentin expression in neural cells of hippocampus DG at amplifying 400. **C** Representative images of vimentin expression in neural cells of cingulate cortex at amplifying 200. **D** Representative images of vimentin expression in neural cells of cingulate cortex at amplifying 400. All vimentin expressed in the extranuclear. Representative images of vimentin and Brdu double labeled immunofluorescent staining in cerebrum **(E, F)**.** E** Representative image of vimentin and Brdu double labeled immunofluorescent staining in LV at amplifying 200 in WT mice. **F** Representative images of vimentin and Brdu double labeled immunofluorescent staining in the hippocmpus at amplifying 200 in WT mice. In the cerebrum, only less than 20% of VPCs were doubly labeled by Brdu in LV and hippocampus, other regions almost weren't found their double labeling. Results indicated that only fewer VPCs have the proliferation ability. Distribution features of VPCs in the different regions of cerebrum at the different stages in adult WT and Tg(SOD1*G93A)1Gur mice (G-I).** G** The comparison of VPCs number in different regions of cerebral cortex at different stages in Tg(SOD1*G93A)1Gur mice. The VPCs number significantly decreased following with the progression of disease from pre-onset, onset to progression stages in the olfactory, the cingulate and the motor cortex compared with controls. The VPCs number didn't significantly change in the sensory cortex. **H** The comparison of VPCs number in different regions of cerebrum at different stages in WT mice. The VPCs number didn't significantly change in different regions of cerebrum at different stages in WT mice. **I** The comparison of VPCs number in different regions of LV and hippocampus at different stages in Tg(SOD1*G93A)1Gur mice. The VPCs number in LV and hippocampus didn't significantly change at onset and progression stages of Tg(SOD1*G93A)1Gur mice compared with controls. Results indicated that only VPCs number in the cerebral cortex significantly decreased following with the disease progression, other regions didn't significantly change. Control mice were age-matched non-transgenic normal C57BL/6 mice, their age were 60-70 days, 90-100 days and 120-130 days respectively. WT mice were C57BL/6 SOD1 wild-type mice.

Vimentin positive cells (VPCs) were found to extensively exist in many regions of cerebrum, which were mainly distributed in the ventricular system, periventricular structures, the hippocampus and the cerebral cortex (Fig. 1A, B), among them, the number and density of VPCs exited the significant difference in different cerebral regions. The number of VPCs was that in cerebral ventricle systems more than that in the cerebral cortex more than that in periventricular structures more than that in the hippocampus (e.g. ventricles systems > cerebral cortex > periventricular structures > hippocampus) in the cerebrum (Fig. 1A). The percentage of VPCs was the ventricular system (99±0.9%) > periventricular structures (47±5%) > the hippocampus (23±2%) > the cerebral cortex (6±1%) in the cerebrum (Fig. 1B). In the cerebral ventricular system, almost all epithelial cells in the interior side of ependyma were VPCs. The cell number of VPCs was LV > Aq > 4V > 3V > D3V > CC (Fig. 1C, Fig. 2). In the periventricular structure, the cell number of VPCs was periventricular LV > periventricular D3V > periventricular 3V (Fig. 1D, Fig. 2), LS > EC > IC > MHb > PH (Fig. 3A, Fig. 4). In the hippocampus, the cell number of VPCs was CA1 > LMol > DG > CA2 > CA3 (Fig. 3B, Fig. 5). The percentage of VPCs was LMol (57±3%) > CA1 (27±3%) > CA2 (20±2%) > DG (12±2%) > CA3 (4±1%) (Fig. 3C, Fig. 5). In the CA1, VPCs were mainly located in the Or and the Rad, the distribution in the Py was very fewer. In the cerebral cortex, VPCs located in almost all cerebral cortex. The number of VPCs was the olfactory cortex > the cingulate cortex > the sensory > the motor cortex (Fig. 3D, Fig. 6A-D). The percentage of VPCs was the olfactory cortex (24±3%) > the cingulate cortex (19±2%) > the motor cortex (4±0.9%) > the sensory cortex (3±0.09%) (Fig. 3E, Fig. 6A-D). There was no significant difference in the number of VPCs in left and right brains, and no significant difference in the normal mice at different ages (60-130 days). The major VPCs in the cerebrum overlapped with GFAP staining, indicating almost all VPCs were astrocytes (Fig. 6E, F). All vimentin expressed in the extranuclear (Fig. 7A-D).

### Distributed features of Brdu positive cell in the adult cerebrum of WT and Tg(SOD1*G93A)1Gur mice

Brdu positive cells were mainly distributed in the lateral ventricle and the hippocampus in the cerebrum. In the lateral ventricle, it mainly located in epithelial cells in the exterior side of lateral ventricle. In the hippocampus, VPCs mainly located in the granular lower lay of DG. There was no significant difference in the number of Brdu positive cells in the lateral ventricle and the hippocampus between WT mice and Tg(SOD1*G93A)1Gur mice and among different periods of Tg(SOD1*G93A)1Gur mice (Fig. 7E, F). The distribution of Brdu positive cells was consistent with that of VPCs. The result showed that VCCs in the cerebrum didn't proliferate in the ALS-like Tg(SOD1*G93A)1Gur mice.

### Distributed features of VCCs in the adult cerebrum of Tg(SOD1*G93A)1Gur mice

**Figure 8:**
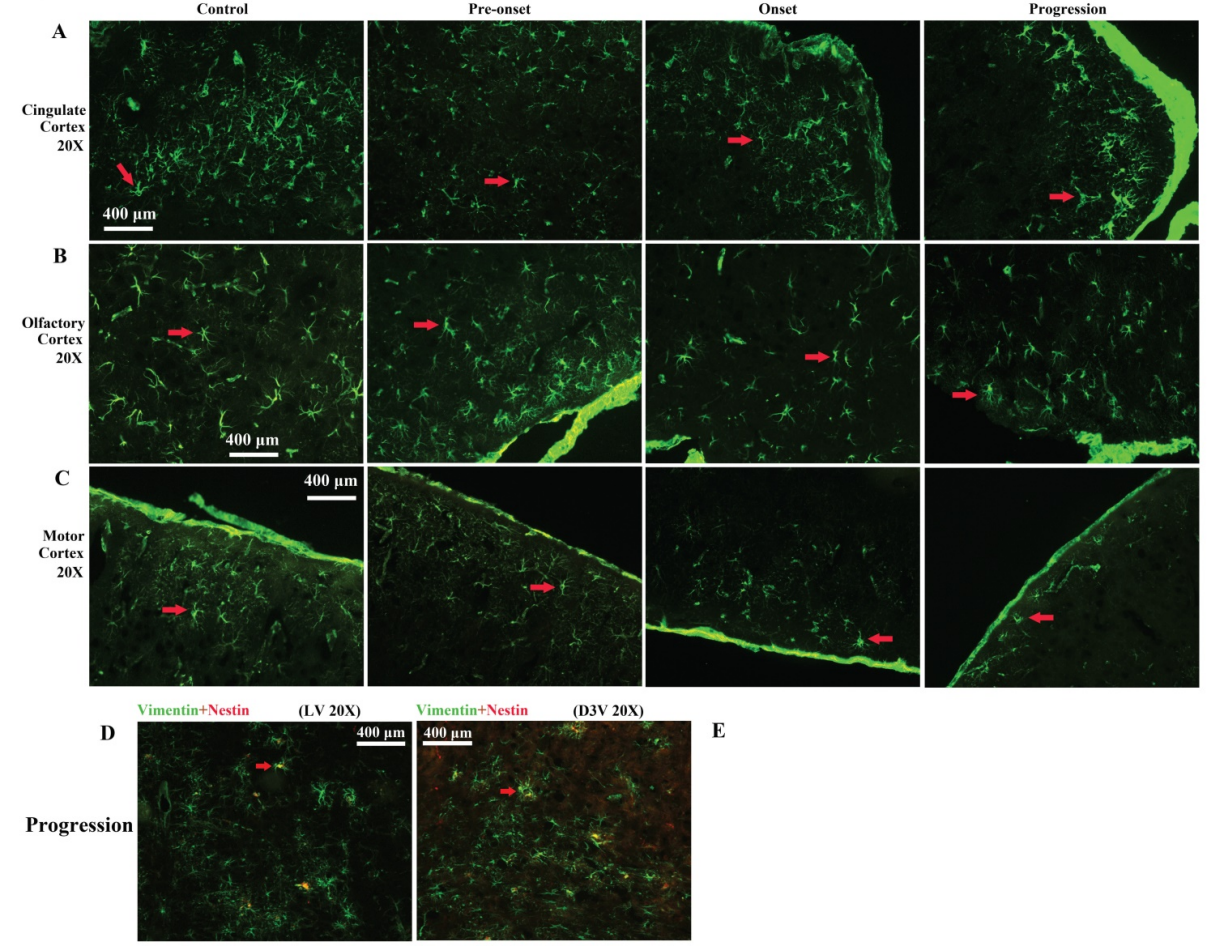
Representative images of VPCs distribution in different regions of cerebral cortex at different stages in Tg(SOD1*G93A)1Gur mice. A Representative images of VPCs distribution in the cingulate cortex at different stages in Tg(SOD1*G93A)1Gur mice. The VPCs number and density significantly reduced from pre-onset, onset to progression stages compared with controls. **B** Representative images of VPCs distribution in the olfactory cortex at different stages in Tg(SOD1*G93A)1Gur mice. The VPCs number and density significantly reduced from pre-onset, onset to progression stages compared with controls. **C** Representative images of VPCs distribution in the motor cortex at different stages in Tg(SOD1*G93A)1Gur mice. The VPCs number and density significantly reduced from pre-onset, onset to progression stages compared with controls. Representative images of vimentin and nestin double labeled immunofluorescent staining in the cerebrum **(D, E)**.** D** Representative image of vimentin and nestin double labeled immunofluorescent staining in the around LV at amplifying 200 in the progression stage of Tg(SOD1*G93A)1Gur mice. **E** Representative image of vimentin and nestin double labeled immunofluorescent staining in the around D3V at amplifying 200 in the progression stage of Tg(SOD1*G93A)1Gur mice. In the cerebrum, only less than 30% of VPCs were doubly labeled by the nestin in LV and D3V.

In the cerebral cortex of Tg(SOD1*G93A)1Gur mice, VPCs significantly reduced in the motor cortex, the olfactory cortex and the cingulate cortex (Fig. 7G), other regions hadn't any significant changes (Fig. 7H, I, Fig. 8A-C). All vimentin expressed in the extranuclear (Fig. 7A-D). Results implied that VCCs might migrate to other brain regions, and no any redistribution in neural cells occurred in the ALS-like Tg(SOD1*G93A)1Gur mice.

### Distributed features of nestin positive cell in the adult cerebrum of WT and Tg(SOD1*G93A)1Gur mice

All nestin positive cells were expressed vimentin, nestin positive cells only occupied approximate 24% of VPCs. There was no significant difference in the number of nestin positive cells in the cerebrum between WT mice and Tg(SOD1*G93A)1Gur mice and among different periods of Tg(SOD1*G93A)1Gur mice (Fig. 8D, E). Results indicated that partial VCCs were NPCs, and further determined NPCs in the cerebrum didn't proliferate in the ALS-like Tg(SOD1*G93A)1Gur mice.

## Discussion

In this study, we obtained following findings through observing and analyzing altered features in different anatomic regions and different neural cells of cerebrum at different disease stages of Tg(SOD1*G93A)1Gur mice and matched same time points of WT mice. 1) In normal adult mice, the cerebrum extensively existed vimentin-containing cells (VCCs), the from more to less rank of VCCs number was ventricular systems more than the cerebral cortex more than periventricular structures more than the hippocampus (Ventricular systems > cerebral cortex > periventricular structures > hippocampus), the from more to less rank of VCCs density (based on the percent of VPCs in local regions) was ventricular systems more than the cerebral cortex more than the hippocampus more than periventricular structures (Ventricular systems > cerebral cortex > hippocampus > periventricular structures). The regions of VCCs mainly located in the lateral ventricle and hippocampus. Partial VCCs were NPCs or NSCs. 2) In ALS-like Tg(SOD1*G93A)1Gur mice, VCCs in the motor cortex, the olfactory cortex and the cingulate cortex showed that no any proliferation and redistribution of VCCs in neural cells of cerebrum occurred in all stages of ALS.

### Alteration of VCCs distribution in the cerebrum of Tg(SOD1*G93A)1Gur mice

Compared with WT mice, the VCCs distribution in the entire cerebrum of Tg(SOD1*G93A)1Gur mice gradually reduced accompanying with the progression of disease, especially significant regions were in the cerebral cortex such as the motor, olfactory and cingulate cortex. Vimentins are class-III intermediate filaments found in various non-epithelial cells, especially mesenchymal cells. Vimentin is attached to the nucleus, express in the extranuclear including the endoplasmic reticulum, and the mitochondria, either laterally or terminally at the normal condition (Fig. 7A-D) 53. Nestin is a type VI intermediate filament protein, are expressed mostly in the axon of nerve cells, the nestin expression has been extensively used as a biomarker of NPCs or NSCs in the CNS 54, 55. In the CNS of adult mammalian animal, vimentin exits in the late stage of differentiating neural cell after the nestin protein almost disappears, is a biomarker of NPCs or NSCs of differentiated late stage 45-48. In our experimental result, partial VCPs expressed nestin and all nestin positive cells were VCPs (Fig. 8D, E), it indicates that partial VCCs were NPCs or NSCs. We didn't detect VCCs increase in the entire cerebrum of Tg(SOD1*G93A)1Gur mice, which indicated that NPCs or NSCs didn't increase at all stages of ALS. Thus, we suggested that a potential neuroregenerative response didn't occur in the cerebrum of Tg(SOD1*G93A)1Gur mice, and the ability of neuroregeneration gradually attenuated with the disease progression of ALS. It also implied that the repairing ability in the cerebral cortex was very limited and weak in ALS-like Tg(SOD1*G93A)1Gur mice. In other words, the cerebral cortex almost hasn't the self-recover function in ALS-like Tg(SOD1*G93A)1Gur mice.

### Alteration of VCCs proliferation in the cerebrum of Tg(SOD1*G93A)1Gur mice

After injected Brdu into animal in vivo, cells were stained by the anti Brdu monoclonal antibody; positive cells are considered as proliferating cells 56. In WT mice, Brdu positive cells mainly exited in the lateral ventricle and the hippocampus of cerebrum, thus, our studied results indicated that the neuroregeneration mainly occurred in the lateral ventricle and the hippocampus of cerebrum in normal adult mice, which were consistent with previous reported studied results 39, 40, 42-44. However, VCCs doubly stained by Brdu were not found to increase at any regions and any stages, which indicated that the proliferation of VCCs didn't occur in the cerebrum of Tg(SOD1*G93A)1Gur mice. Thus, we suggested that it didn't trigger the proliferation of NPCs or NSCs in the cerebrum, also implied no self-repair ability in the cerebrum of ALS-like Tg(SOD1*G93A)1Gur mice.

### Alteration of VCCs differentiation in the cerebrum of Tg(SOD1*G93A)1Gur mice

Current studies found that NPCs or NSCs in the brain and the spinal cord extensively proliferated, differentiated and migrated damaged regions to restore damaged neural cells at some pathological conditions such Parkinson's disease, Alzheimer's disease, spinal cord injury and ALS, partial NPCs or NSCs differentiated into astrocytes, oligodendrocytes and neuron cells, but the self-repair ability was very limited 39, 40, 42, 52, 57. However, in our study, the number and density of VCCs in the cerebrum of Tg(SOD1*G93A)1Gur mice weren't found to significantly increase at all stages of ALS-like Tg(SOD1*G93A)1Gur mice compared with WT mice. All VCCs were astrocytes in both WT and Tg(SOD1*G93A)1Gur mice because all VCCs were GFAP positive cells 58. VCCs in the cerebrum didn't differentiate into other neural cells in ALS-like Tg(SOD1*G93A)1Gur mice.

### Alteration of VCCs migration in the cerebrum of Tg(SOD1*G93A)1Gur mice

Our results showed that VCCs in the motor cortex, the olfactory cortex and the cingulate cortex significantly reduced and the number and density in the entire cerebrum didn't significantly increase. Previous studies reported that NPCs or NSCs in reduced regions of CNS migrated to damaged regions to restore neural cells at some pathological conditions 39, 40, 42, 52, 57. Therefore, we hypothesized that reduced VCCs in the motor cortex, the olfactory cortex and the cingulate cortex might migrate to other brain regions, and play a self-restoring ability to repair damaged neural cells in ALS-like Tg(SOD1*G93A)1Gur mice. However, our objective experimental results didn't observe and detect the migration of VCCs. The alteration of VCCs migration in the cerebrum of Tg(SOD1*G93A)1Gur mice only was one hypothesis based on other previous studied reports. If the amount of VCCs decreased in the cortex, it was also highly possible that they were not migrating to other places but merely decrease in situ due to the disease itself. Alteration of VCCs migration in the cerebrum of Tg(SOD1*G93A)1Gur mice need provide direct evidences and results support this inference of VCCs migration in the study.

Endogenous NPCs and NSCs are more and more valued by investigators following with the finding of NPCs or NSCs exist in the CNS of adult mammal animal 39-41, 42-44. Recent studies showed that endogenous NPCs or NSCs could proliferate, differentiate and directly migrate to damaged brain regions in some pathological conditions, such as Parkinson's disease 39, Alzheimer's disease 42, spinal cord injury 41 and ALS 40, exert the limited self-repair effect, but couldn't effectively stop the progression of these disease. If the proliferate, differentiate and directly migrate of endogenous NPCs or NSCs could been modulated or enhanced at some pathological conditions in vivo, it would bring the wonderful hope to treat some diseases of CNS lesion like ALS through enhancing the repair ability of endogenous NPCs or NSCs, and avoid a lot of side effects caused by the transplantation of exogenous NPCs or NSCs. However, up to now, any effective methods to modulate or enhance their proliferation, differentiation and direct migration in vivo haven't been found yet. Thereby, it is very important to search the pathway regulating their ability of proliferation, differentiation and direct migration in the pathological condition like ALS, obtain the effective treatment of ALS. This study is a preliminary study modulating the proliferation, differentiation and direct migration of NPCs and NSCs in vivo. Our results will provide some evidences and clues for further modulating them in the pathological condition like ALS. In general, NPCs or NSCs extensively exist in the adult cerebrum.

## Limitations and prospects

Vimentin was used as a marker to label NPCs in the cerebrum in this study. It was critical to evaluate whether vimentin labeled all NPCs or only part of NPCs? In fact, the vimentin expression will reduce and even disappear when progenitor cells and/or NPCs terminally differentiate into neurons or glial cells, vimentin mainly expresses at the later stage of NPCs. In addition to vimentin, other NPCs markers (Like nestin, nestin labels NPCs of earlier stages. DCX) and proliferation markers (Like Ki67) also need to be examined. Moreover, whether the expression of nestin+ or DCX+ cells is altered at different stages of ALS mice will further delineate the changes of NPCs in the ALS mouse model. GFAP was used as a marker for astrocyte. However, GFAP is also expressed by radial glial cells (NPCs). Other astrocyte markers (Like Ki67) need to be used to confirm whether they are astrocytes or progenitor cells or NPCs. All vimentin expressed in the extranuclear, almost all VCCs were astrocytes in WT mice and Tg(SOD1*G93A)1Gur mice. Thus, if VCCs were isolated from the cerebrum of WT and ALS mice and then to study what cells types they could become in our study, which will more objectively observe the differentiation alteration of NPCs in the cerebrum between WT and ALS mice. Therefore, if above described experiments could be finished in our study, our study would become more perfect. These deficiencies in our study will be the best idea and direction of our further study.

## Abbreviations

ALS: amyotrophic laterale sclerosis
VCCs: vimentin-containing cells
GFAP: glial fibrillary acidic protein
NeuN: hexaribonucleotide Binding Protein-3
Oli-2: anti-oligodendrocyte antibody-2
Ox-42: Integrin αM
DAPI: 4',6-diamidino-2-phenylindole
OB: Olfactory bulb
Gl: glomerular layer
AOB: accessory OB
GRO: granular cll layer
MI: mitral cell layer
IPL: internal plexiform layer
CC: central canal
LV: lateral ventricle
PS: periventricular structure
3V: 3rd ventricle
4V: 4th ventricle
D3V: dorsal 3rd ventricle
Aq: aqueduct (Sylvius)
EC: external capsule
IC: internal capsule
LS: lateral septal nucleus
MHb: medial habenular nucleus
PH: posterior hypothalamic area
LMol: lacunosum moleculare layer of the hippocampus
DG: dentate gyrus
CA1: field CA1 of hippocampus
CA2: field CA2 of hippocampus
CA3: field CA3 of hippocampus
Or: oriens layer of the hippocampus
Rad: stratum radiatum of the hippocampus
Py: pyramidal cell layer of the hippocampus
Sl: subgranular layer of the hippocampal DG
GI: gigantocellular reticular nucleus
ICP: inferior cerebellar peduncle (restiform body)
PnC: pontine reticular nucleus, caudal part
PnO: pontine reticular nucleus, oral part
Vesti: Vestibular nucleus
7N: facial nucleus, and facial nerve or its root
LC: locus coeruleus
Sol: nucleus of the solitary tract
MdD: medullary reticular nucleus, dorsal part
MdV: medullary reticular nucleus, ventral part
IRt: intermediate reticular nucleus
Cu: cuneate nucleus
PAG: periaqueductal gray
